# Tibiofemoral joint contact area and stress after single-bundle anterior cruciate ligament reconstruction with transtibial versus anteromedial portal drilling techniques

**DOI:** 10.1186/s13018-018-0956-1

**Published:** 2018-10-04

**Authors:** Chunhui Liu, Yingpeng Wang, Zhongli Li, Ji Li, Hao Zhang, Yangmu Fu, Kuan Zhang

**Affiliations:** 1 Department of Orthopedics, General Hospital of PLA, No. 28 Fuxing Road, Haidian District, Beijing, 100853 China; 2 School of Biomedical Engineering, Capital Medical University, Beijing, 100069 China

**Keywords:** Anterior cruciate ligament reconstruction, Transtibial technique, Anteromedial portal technique, Tibiofemoral, Contact area, Contact stress

## Abstract

**Background:**

During single-bundle ACLR, femoral tunnel location plays an important role in restoring the intact knee mechanisms, whereas malplacement of the tunnel was cited as the most common cause of knee instability. The objective of this study is to evaluate, objectively, the tibiofemoral contact area and stress after single-bundle (SB) anterior cruciate ligament reconstruction (ACLR) with femoral tunnel positions drilled by transtibial (TT) or anteromedial (AM) portal techniques.

**Methods:**

Seven fresh human cadaveric knees underwent ACLR by the use of TT or AM portal techniques in a randomized order. These specimens were reused for ACL-R (TT and AM). The tibiofemoral contact area and stresses were gauged by an electronic stress-sensitive film inserted into the joint space. The knee was under the femoral axial compressive load of 1000 N using a biomechanics testing machine at 0°, 10°, 20°, and 30° of flexion. Three conditions were compared: (1) intact ACL, (2) ACLR by the use of the TT method, and (3) ACLR by the use of the AM portal method.

**Results:**

Compared with AM portal ACL-reconstructed knees, a significantly decreased tibiofemoral contact area on the medial compartment was detected in the TT ACL-reconstructed knees at 20°of knee flexion (*P* = .047). Compared with the intact group, the TT ACLR group showed a higher mean stress at 20° and 30° of flexion on the medial compartments (*P* = .001, *P* = .003, respectively), while the AM portal ACLR group showed no significant differences at 30° of flexion (*P* = .073). The TT ACLR group also showed a higher mean maximum stress at 20° of flexion on the medial compartments (*P* = .047), while the AM portal ACLR group showed no significant differences at this angle(*P* = .319).

**Discussion:**

The alternation of the tibiofemoral joint contact area and stress in reconstructed knees may be caused by the mismatch of the tibiofemoral joint during knee movement procedures compared with intact knees.

**Conclusions:**

SB ACLR by the use of the AM portal method and TT method both alter the tibiofemoral contact area and stress when compared with the intact knee. When compared with the TT technique, ACLR by the AM portal technique more closely restores the intact tibiofemoral contact area and stress at low flexion angles.

## Background

Among the current methods of anterior cruciate ligament reconstruction (ACLR), single-bundle(SB) reconstruction is performed by most surgeons [1, 2]. During single-bundle ACLR, femoral tunnel location plays an important role in restoring the intact knee mechanisms, whereas malplacement of the tunnel was cited as the most common cause of knee instability [3–5]. As a result, the best location of the femoral tunnel during single-bundle ACLR is subject to extensive exploration with the development of anatomic studies [6–9]. There are mainly two methods for femoral tunnel creation: transtibial versus anteromedial portal techniques. The current femoral tunnel preparation focus has shifted from the TT method(with femoral tunnel location at the “over-the-top” position approximately 11 o’clock in the femoral notch of the right knee) toward the independent drilling method(with the femoral tunnel location at the center of the AM bundle of the ACL footprint approximately 9 o’clock in the femoral notch of the right knee) with restoration of the native ACL knee kinematics. The use of the AM portal drilling technique has increased in recent years from 68% of surgeons using this technique in 2013 [1] to 89.6% in 2016 [2]. Although the anatomic single-bundle ACLR procedure is currently in use, it remains controversial whether the AM technique is biomechanically superior compared with the TT method. Investigators focusing on the femoral tunnel position have shown improvements in knee stability by placing the femoral tunnel into the native femoral footprint [5, 10–12]. However, not all of the results supported the advantages of anatomical reconstruction. Other studies have demonstrated that no significant knee kinematic changes were found between the TT versus AM portal drilling techniques [13–15].In addition, two meta-analyses showed that there were no significant clinical differences found between the two techniques [13, 16]. As a result of these conflicting outcomes, the best technique of femoral tunnel creation for restoring intact knee kinematics remains unclear. Therefore, the purposes of this study were (1) to quantify the effect of two femoral tunnel creation methods on the tibiofemoral joint contact area and stress after single-bundle ACL reconstruction, (2) to identify the optimal femoral tunnel creation method, and (3) to give new evidence to the present conflicting results. The hypothesis was that SB ACLR by the use of the AM portal method would better restore the intact tibiofemoral contact area and stress compared with the TT method.

## Methods

### Preparation of cadaveric knees

**Fig.1:**
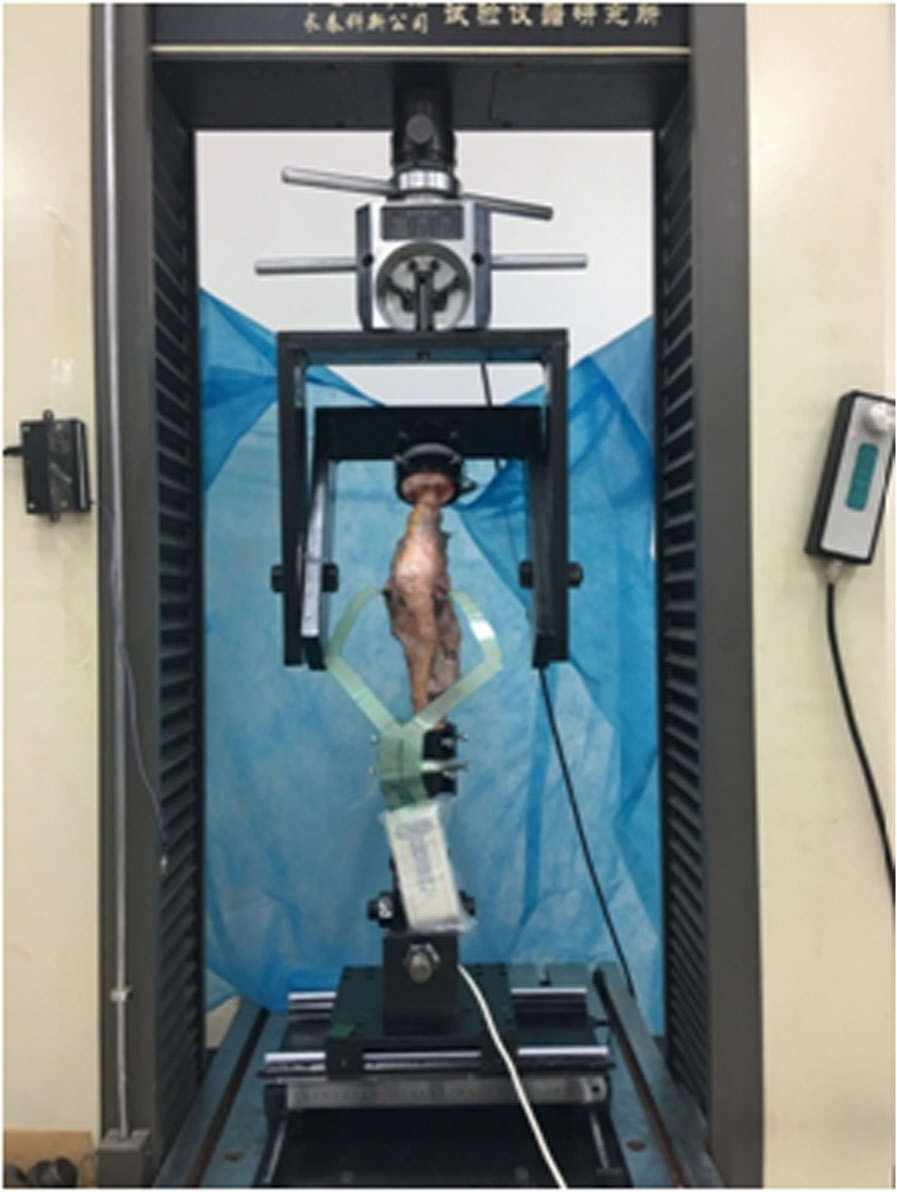
A biomechanical testing machine and a knee simulator

Seven intact fresh-frozen human cadaveric knees (mean age, 58 years; range, 46 to 71 years,4 males and 3 females) without macroscopic degenerative changes were used in this study. Lateral and anteroposterior X-ray films were taken for each knee with the aim of assessing signs of osteoarthritis or bony deformities. No cadaveric knees were excluded from the study. The specimens were stored in sealed plastic bags at − 20 °C and thawed 24 h at room temperature when they were prepared to be tested. The gracilis and semitendinosus tendons were harvested from each knee for ACL reconstruction. The proximal tibia and distal femur were then cut approximately 20 cm from the joint line. The skin and all subcutaneous tissues were removed, leaving all but the posterior portion of the joint capsule, with the cruciate and collateral ligaments intact. The soft tissues 13 to 15 cm away from the joint line were subsequently cut off so that the proximal and distal bones were exposed not less than 5 cm [5]. The proximal and distal bone stumps were then embedded in custom-made plastic cylinders using acrylic resin polymer (Anyang Eagle Dental Material Co., Ltd., Products, China, Anyang City). The femoral cylinder was fixed to the top of the testing machine using custom-made fixtures, while the tibial cylinder was connected to a specially designed knee simulator that allows 6 degrees of freedom of movement of the knee (anterior-posterior, medial-lateral, and internal rotation-external rotation) (Fig. 1).

### Normal gait simulator

For this study, combined external conditions simulating normal gait were applied to the knee: (1) considering that some of the specimens were skinny, an axial load of 1000 N was applied by a biomechanical testing machine (Electronic Universal Material Testing Machine, WDW4100, China, Changchun City) from the femoral side to the tibia along the direction of the tibial longitudinal axis (2) at 0°, 10°, 20°, and 30°of knee flexion. This protocol was based on the work of Kumar et al. and Kutzner et al. [17, 18] in which the authors analyzed the tibiofemoral reactive stress that the knee experiences during normal gait. The custom-designed knee simulator allows 6 degrees of freedom of movement of the knee that can simulate the normal gait closely. For knee flexion, the flexion angles were achieved by moving the femur on the sagittal plane locked in position by two screw fixtures at each side of the normal gait simulator. The axial testing machine and knee simulator helped to achieve these goals perfectly (Fig. 1).

### Tibiofemoral contact area and stress measurement

**Fig. 2:**
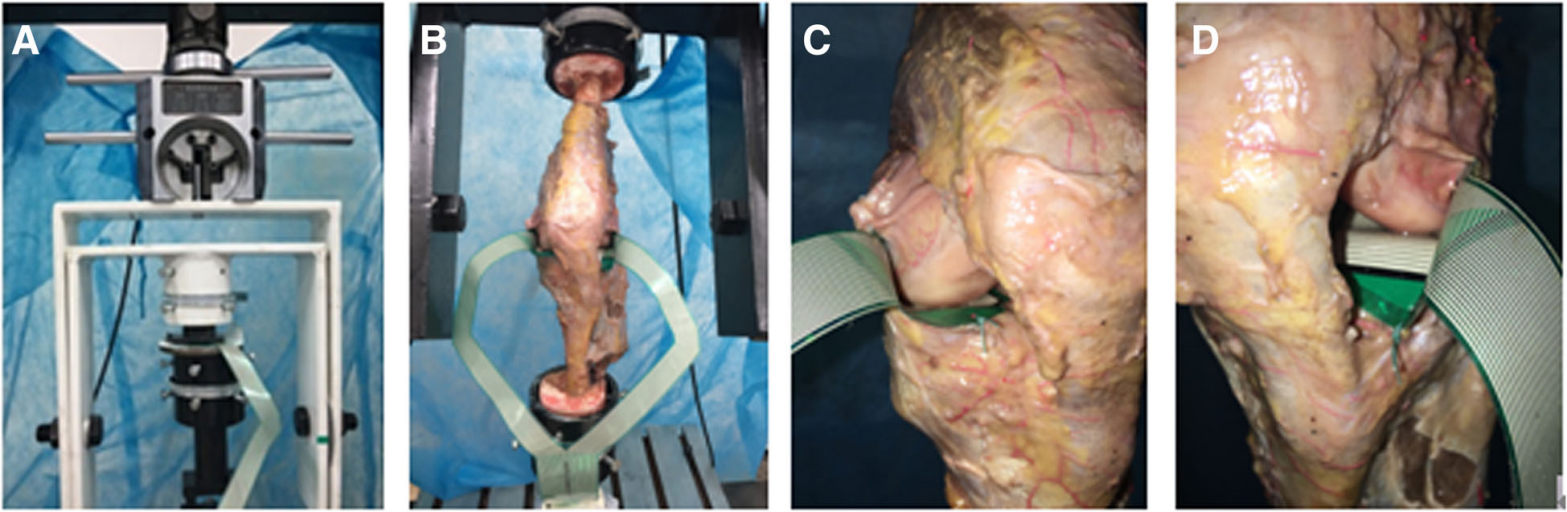
The experimental testing system (a sensor was calibrated according to the standardized protocols provided by the manufacturer (**a**), a left knee mounted onto the machine (**b**), a film was carefully inserted into the joint and then spread on top of the cartilage and the meniscus (**c**, **d**)) presented in this study with a left knee

Stress-sensitive film (K-Scan 4000, Tekscan Inc., Boston, MA) of 0.1 mm was used in this system. Before insertion into the joint spaces, the sensors were calibrated according to the standardized protocols provided by the manufacturer [19]. An incision between the meniscus and femoral condyle was made along the joint line through the anteromedial and anterolateral arthrotomy. The film was then carefully inserted into the joint and then spread on top of the cartilage and the meniscus [20]. The contact area and peak and mean stress in the tibiofemoral joint were measured at the knee flexion angles of 0°, 10°, 20°,and 30° combined with axial load simulating the joint motion during normal walking [21]. The knees were loaded axially for 20 cycles to simulate various phases of the gait cycle for each testing condition and flexion angle. To assess changes in the tibiofemoral contact area and stress, the same external conditions previously applied to the intact knees were again applied to the ACLR knees, and the results were measured. The experimental testing system is shown in Fig. 2.

### Surgical procedures and testing groups

#### Graft preparation and fixation

The distal 3 cm of the semitendinosus and gracilis tendon grafts were stitched by a polyester thread (No. 5 TiCron suture, Covidien plc, Dublin, Ireland) and placed on a tensioning board under the tension of 10 N for 15 min. Subsequently, a 15- to 30-mm EndoButton CL was used to suspend at the middle of the semitendinosus and gracilis tendons, and then the grafts were folded into four bundles with a diameter of 7–8 mm. The graft in the femoral tunnel was suspended fixed by the use of the EndoButton CL (Smith & Nephew Endoscopy). After 10 flexion-extension cycles under 80 N of graft tension, the graft fixation at the tibial side was accomplished under 30° of knee flexion with the maintained tension [22] by the use of an interference screw (Arthrex, Naples, FL). The interference screw diameter was 1 mm up from the graft. The graft was then released by loosening the tibial screw after biomechanical testing of the first reconstruction, and the first femoral tunnel was backfilled with bone cement (Link, Germany). The alternate femoral tunnel was then drilled, partially overlapping the first tunnel occasionally. The two methods of femoral tunnel creation were performed in an alternating order, with the aim of randomizing the possible influences of previous reconstructions [22]. The graft fixation methods were subsequently repeated to perform the second reconstruction with the same graft with one exception: previous screws were substituted for 1-mm up screws in the tibia to compensate for any bone tunnel deformity that may have occurred during the initial reconstruction [23]. We used the same graft for the limited source of the tendon. We placed the tendons on a tensioning board under the tension of 10 N for 15 min in order to reduce the difference caused by reuse.

#### Tibial tunnel preparation for the initial reconstruction

The tibial tunnel was placed using a commercial tibial ACL guide (Smith & Nephew, ACUFEX DIRECTOR™ Drill Guide) set at 55°with the tip aimed at the center of the tibial footprint of the ACL and the sleeve positioned at the midpoint of the anterior margin of the medial collateral ligament and the medial margin of the tibial tubercle [24]. A K-wire was drilled into the tibia along the ACL guide, and a tibial tunnel was then reamed over by the use of a cannulated drill along the K-wire. The tunnel diameter was finally created with regard to the graft diameter prepared previously.

#### SB-TT technique group

For the TT method of ACL reconstruction, the inside entrance of the femoral tunnel was located using an offset guide (EndoButton Guide; Smith & Nephew), which was passed through the tibial tunnel and hooked at the “over-the-top” position, assuring that the posterior edge of the femoral tunnel was placed 2 mm anterior from the posterior edge of the intercondylar notch. With the aim of positioning the guidewire for the most possible approximation of the anatomic femoral ACL footprint, the offset guide was then laterally rotated [24]. After the desirable position was located, a guidewire was drilled into the femur, and then a 4.5-mm-diameter cannulated drill (EndoButton Drill; Smith & Nephew Endoscopy) was reamed along the guidewire until it pierced through the femoral cortex. The length of the graft inserted into the tunnel was not less than 15 cm and was decided by the total femoral tunnel length measured by a depth probe (Depth Probe; Smith & Nephew Endoscopy). A blunt head reamer was then used to create a graft diameter femoral tunnel with 10 mm more than the inserted graft length. The graft fixation technique described previously was used finally to accomplish the SB-ACLR.

#### SB-AM portal technique group

The AM portal method was performed by the use of an offset guide inserted through the independent AM portal. The hook of the femoral offset guide was placed behind the posterior notch and adjusted in order to place the pointer at the center of the femoral AM bundle footprint of the ACL at 90°of knee flexion. The knee was then flexed to 110°, and a 2.4-mm guidewire was drilled into the lateral condyle with the offset guide. The femoral tunnel preparation for EndoButton fixation was then accomplished, as described above. The graft fixation was performed as described previously.

The testing groups were as follows: intact knee group, SB-TT technique reconstruction group and SB-AM portal technique reconstruction group. These specimens were reused for ACL-R (TT and AM).

### Statistical analyses

Because all variables were measured within each specimen, the tibiofemoral contact area and stress data were analyzed using a two-way analysis of variance (SPSS, version 17.0 Chicago, IL). This analysis has the advantage of minimized specimen variability and being very sensitive to relative changes occurring within an individual knee. Multiple contrasts were detected by the post hoc Tukey multiple comparisons test for all experiments performed on the same knee at each knee flexion angle tested. *P* < .05 was set as the level of significance a priori.

## Results

### Tibiofemoral contact area

**Fig. 3:**
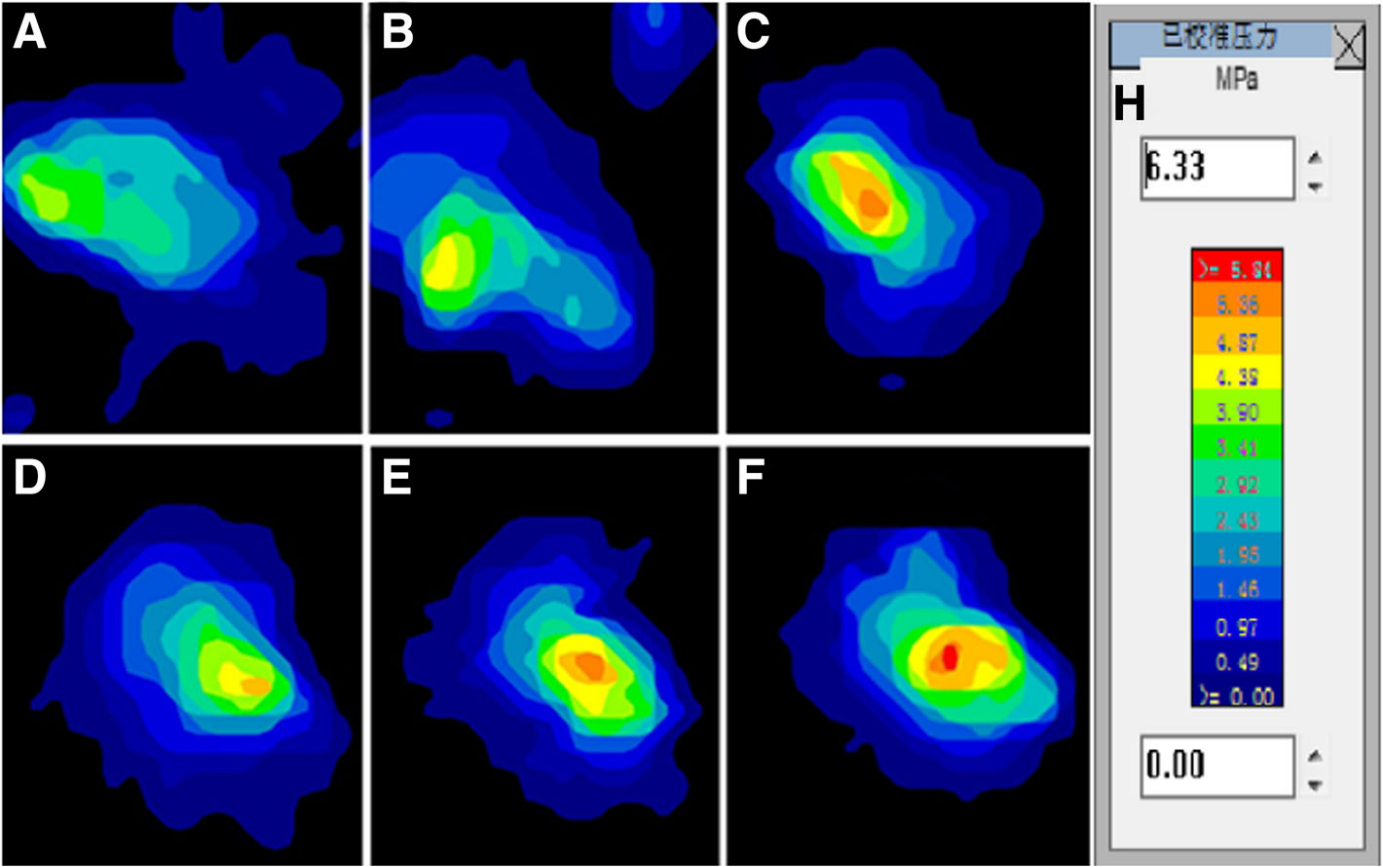
K-Scan 4000 contact area and stress maps representative of a left knee under 1000 N axial load at 20° of flexion after undergoing the two ACLR conditions. Medial tibiofemoral joint of intact knee (**a**), medial joint of AM portal technique reconstructed knee (**b**), medial joint of TT technique reconstructed knee (**c**), lateral joint of intact knee (**d**), lateral joint of AM portal technique reconstructed knee (**e**), lateral joint of TT technique reconstructed knee (**f**). Calibrated contact stress legend (**f**). Top = anterior

Compared with the intact ACL group, the tibiofemoral contact area was decreased in the AM portal ACLR group at 20° and 30° of flexion on both the medial and lateral compartments, respectively (*P* = .004 for medial at 20°, *P* = .014 for medial at 30°, *P* = .001 for lateral at 20°, and *P* = .01 for lateral at 30°). For the TT ACL-reconstructed knees, a significantly decreased tibiofemoral contact area was also observed at 20° and 30° of flexion on both the medial and lateral compartments, respectively(*P* < .001 for medial at 20°, *P* = .001 for medial at 30°, *P* < .001 for lateral at 20°, and *P* = .038 for lateral at 30°). Both the AM portal and TT ACLR groups showed no significant difference from the intact ACL group at 0° and 10°, respectively, of knee flexion. When comparing the contact area between the two ACLR groups, however, a significantly decreased contact area was detected in the TT ACL-reconstructed knees at 20° of knee flexion on the medial compartment (*P* = .047) (Fig. 3). There were no significant differences between the TT and AM portal ACL-reconstructed knees on the medial contact area at other angles of flexion and the lateral contact area at all angles of flexion. The values for the contact area are shown in Table 1.

**Table 1:** Contact area results in intact and two different reconstruction groups

	Contact area (mm^2^) (mean ± SD)					
	Medial tibiofemoral joint			Lateral tibiofemoral joint		
	Intact ACL AM portal technique TT technique			Intact ACL AM portal technique TT technique		
0°	515.29 ± 123.43	467.86 ± 119	461.43 ± 117.62	390.29 ± 99.75	358.71 ± 72.86	363.71 ± 79.64
10°	501.71 ± 105.26	456.29 ± 97.68	432.86 ± 101.89	378.14 ± 102.55	335.29 ± 77.98	326.71 ± 76.22
20°	454.57 ± 104.83	381 ± 79.89*	332.86 ± 76.76*^■^	358.14 ± 70.11	305.43 ± 72.48*	285 ± 57.51*
30°	445.71 ± 103.02	400.43 ± 92.16*	383.29 ± 100.17*	393.86 ± 79.26	353.86 ± 78.81*	362.14 ± 73.23*

Tibiofemoral contact area results in intact and two different reconstruction groups (intact, TT technique, and AM portal technique). Single asterisk (*) denotes the difference between the intact state with other states and square symbol (■) denotes the difference between TT technique and AM portal technique

### Mean tibiofemoral stress

**Fig. 4:**
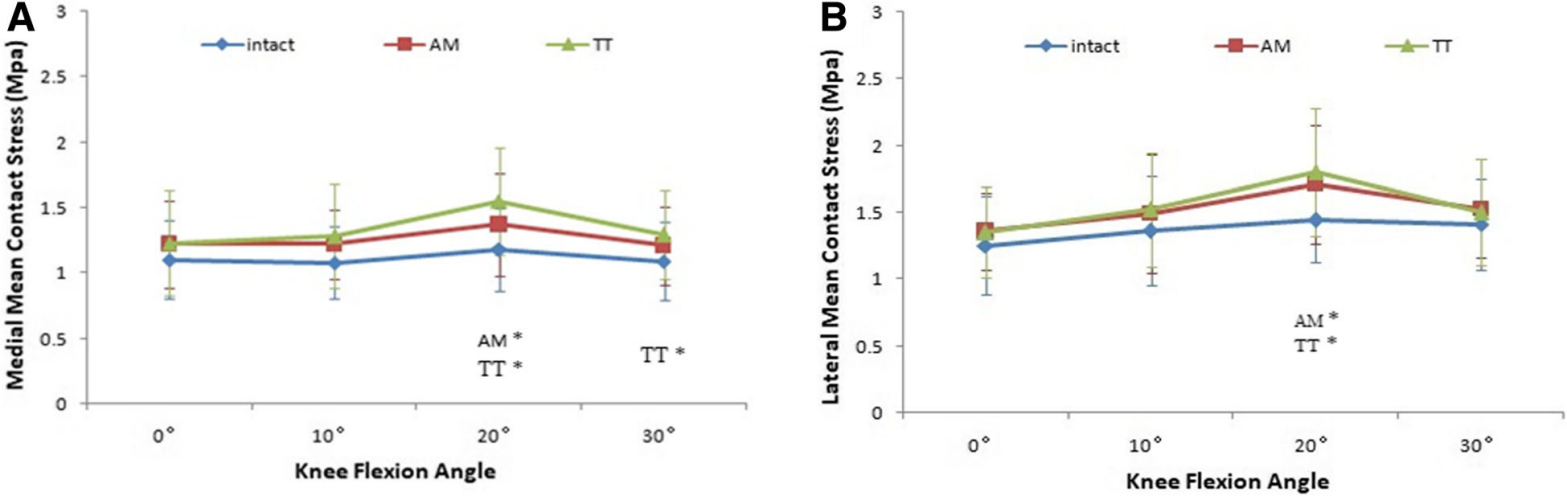
Tibiofemoral mean contact stress (medial mean contact stress (**a**), lateral mean contact stress (**b**)) for each of the three test states (intact, TT technique and AM portal technique). A single asterisk (*) denotes the difference between the intact state and other states

For the AM portal ACL-reconstructed knees, there were no significant differences in mean tibiofemoral stress from the intact knees on both the lateral and medial knee joint compartments, except at 20° of flexion (*P* = .045 for medial and *P* = .006 for lateral). The TT ACLR group showed a higher mean stress at 20° and 30° of flexion on the medial compartment and at 20° of flexion on the lateral compartment compared with the intact ACL group (*P* = .001 for medial at 20°, *P* = .003 for medial at 30°, and *P* < .001 for lateral at 20°), but no significant differences at 0° and 10°of flexion on the medial compartment and at 0°, 10°, and 30° of flexion on the lateral compartment were found. When comparing the mean tibiofemoral stress between the two ACLR groups, however, no significant differences were observed at all angles of knee flexion on both the medial and lateral compartments (Fig. 3). The values for mean stress are shown in Fig. 4.

### Maximum tibiofemoral stress

**Fig. 5:**
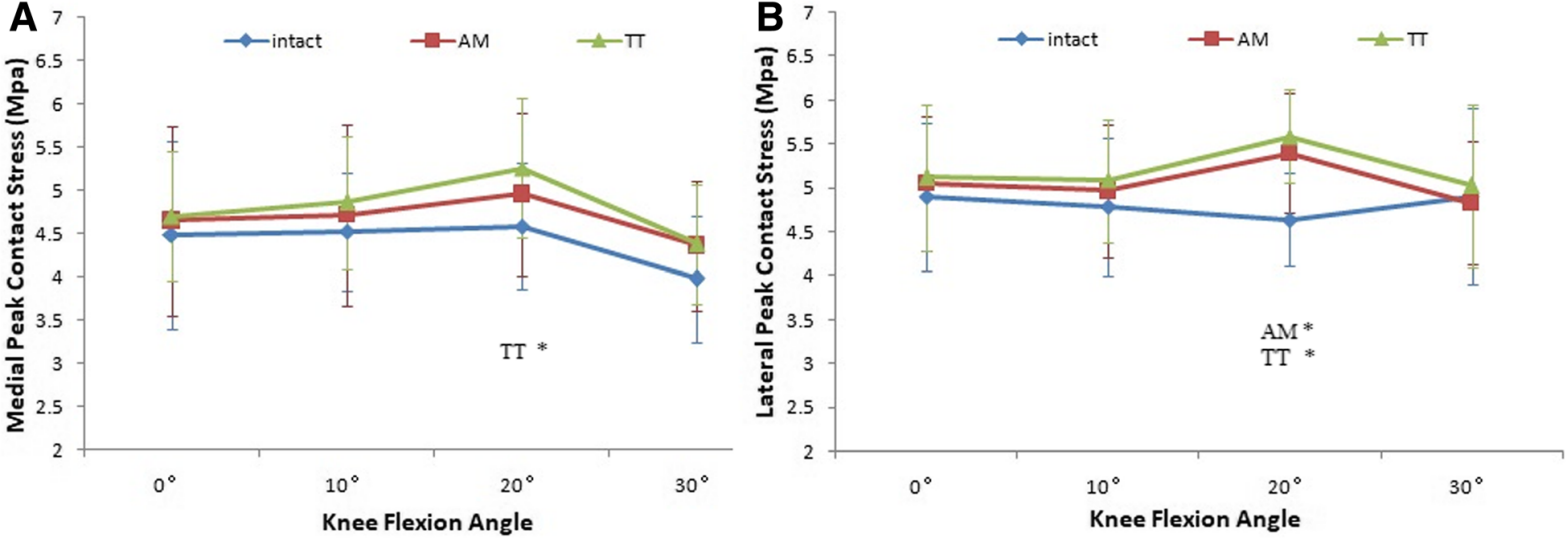
Tibiofemoral peak contact stress (medial peak contact stress (**a**), lateral peak contact stress (**b**)) for each of the three test states (intact, TT technique and AM portal technique). A single asterisk (*) denotes the difference between the intact state and other states

When compared with the intact ACL, the AM portal ACLR altered the maximum stress only on the lateral joint compartment at 20°of flexion(*P* = .022) (Fig. 3). No significant differences were observed at other angles of flexion on the lateral compartment and at all angles of flexion on the medial compartment. For the TT ACL-reconstructed knees, a higher maximum stress was detected at 20° of flexion on both the lateral and medial compartments (*P* = .047 for medial and *P* = .005 for lateral) (Fig. 5). There were no significant differences at all in other flexion angles when compared with the intact ACL knees. Although no significant differences were observed at all angles of knee flexion on both the medial and lateral compartments when comparing the maximum tibiofemoral stress between the two ACLR groups, the results of AM portal ACLR knees were more similar to the intact knees. The values for maximum stress are shown in Fig. 5.

## Discussion

In this study, the tibiofemoral joint contact area and stress of the knees after ACL reconstruction by the AM portal and TT techniques were measured and compared. Specifically, the experimental data collected from the same cadaveric knee specimen under different experimental conditions (ACL-intact and ACL-reconstructed with TT and AMP methods) reduced the effect of interspecimen variation [5]. The results supported that SB ACL reconstruction via the AM portal technique restored the tibiofemoral joint contact area and stress more closely to the intact knee than SB ACL reconstruction via the TT technique. Until now, there have been few studies comparing the changes in tibiofemoral contact mechanics between TT and AM portal ACLR groups to the authors’ knowledge. In one prior study, Lee et al. [25] investigated the contact area and stress in knees with serial posterior medial meniscectomies. In Lee et al., the outcomes of contact area, mean contact stress, and peak contact stress, which were measured in intact knees, were similar to our studies. Another prior study has evaluated the effects of SB ACLR and double-bundle ACLR. In that study, Morimoto et al. [26] tested knees after undergoing SB ACLR and double-bundle ACLR and pointed out that SB ACL reconstruction resulted in a significantly smaller tibiofemoral contact area and higher stress. The peak stress and contact areas measured in their study were also comparable to our data in intact ACL knees, while the mean contact stress reported is higher than our outcomes. This difference may be explained by the type of stress-sensor used in the joint space. And the various experimental conditions and methods for measuring knee contact stress made the comparisons between studies complicated. In their study, the TT or AM portal method was used for femoral tunnel placing. However, in our experience, the femoral tunnel position cannot be placed at the center of the AM bundle position via the TT method. Nevertheless, our study confirms the observation that the SB ACLR condition resulted in decreasing contact area and increasing mean tibiofemoral contact stress and peak contact stress compared with the intact knee.

The alternation of the tibiofemoral joint contact area and stress in reconstructed knees may be caused by the mismatch of the tibiofemoral joint during knee movement procedures compared with intact knees. And the reconstructed ACL, which cannot provide original biomechanics compared with the original ACL, may have resulted in the mismatch of the tibiofemoral joint. Anatomic studies of the ACL indicated that the ligament consists of two grossly distinguishable components: the anteromedial (AM) bundle and posterolateral (PL) bundle [27, 28]. Comparing the in situ forces between the two bundles, the PL bundle has higher in situ forces from full extension to 30° of flexion, whereas the AM bundle has higher in situ forces from 30° of flexion to further flexion under anterior tibial loads [29]. The PL bundle also shows an important role, especially at lower flexion angles under rotatory loads [30]. Such anatomic complexity of the ACL cannot be restored by non-anatomic SB ACLR, which may alter the tibiofemoral joint matching relationship during knee movement procedures and results in a significant alternation of the tibiofemoral joint contact area and stress.

The alternation of the tibiofemoral joint contact area and stress between SB ACL- reconstructed knees via TT versus AM portal drilling techniques may be caused by different femoral tunnel positions. Previous studies indicated that the tunnel location plays an important role in ACL reconstruction, and small variations in the femoral tunnel placement significantly influence the resulting knee kinematics and clinical outcomes [10, 22, 31–33]. Biomechanical studies using cadavers indicated that the AM portal technique placed the femoral tunnel more closely to the anatomic femoral footprint [24, 34, 35], and this may be the reason why SB ACLR by the use of the AM portal method more closely restores the intact tibiofemoral contact area and stress compared with the TT method. There are numerous studies that indicated that the AM portal technique reconstruction provided better rotatory stability at low flexion angles [36–38] without sacrificing anteroposterior stability compared with TT ACL reconstruction [10, 39–42]. Guler et al. [34] and Lee et al. [43] evaluated the femoral tunnel positions created by the AM portal or TT technique in their study and indicated that the AM portal technique is superior to the TT technique in terms of anatomical graft positioning. In a meta-analysis, Riboh et al. [16] reported that there are biomechanical data suggesting improved knee stability and more anatomic graft placement with independent drilling. This literature may also help to explain the superiority of the AM portal technique reconstruction, which better restored the normal knee kinematics, resulting in closer normal contact area and stress, when compared with TT technique reconstruction.

Other studies also have shown that the ACL reconstruction by the use of the TT technique could not effectively prevent the prevalence of secondary knee OA [44–48]. Leiter et al. [49] shown in their meta-analysis that ACL-reconstructed knees using the TT technique had a higher incidence of normal and serious OA than control knees, especially in patients combined with medial meniscus repair or excision. Hart et al. [48] reported in their article that the patellar tender ACL reconstruction using the TT method did not lead to prevention of the occurrence of radiological OA after 10 years by the use of the Kellgren and Fairbank classifications. Janssen et al. [50] found that the radiological signs of OA were detected in 53.5% of the patients with transtibial ACL reconstruction using four-strand hamstring autograft at the 10-year follow-up. However, all of the studies mentioned above were based on the TT technique of ACL reconstruction. With regard to ACL reconstruction using the AM portal method, there are some studies that indicated that anatomic ACL reconstruction showed favorable results regarding OA [51, 52]. Alentorn-Geli et al. [13] indicated in their study that patients in the TT ACLR group had greater long-term knee osteoarthritic changes (greater space narrowing) compared with the AM portal ACLR group when the radiographic parameters were statistically analyzed with a KT-1000 arthrometer. According to Wolff’s law, osteoclasia and bone resorption may be triggered by a low bone stress and an overloading bone stress [53]. Therefore, the ACL reconstructed knee with altered contact area and stress may result in undesirable bone remodeling and predisposition of the knee joint, which finally lead to the occurrence of OA. In this study, ACL reconstruction using the AM portal technique better restored the normal tibial-femoral contact area and stress when compared with the TT technique and may help to explain the favorable results regarding the OA after AM portal technique ACL reconstruction. However, there are too few studies to confirm whether the ACL reconstruction using the AM portal technique will better prevent the occurrence of knee arthritis, and long-term clinical follow-up studies are necessary to verify our hypothesis.

### Limitations

As for the limitations of this study, the number of cadaveric knees used in the experiment was relatively limited, and the donor age and specimen tissue quality were variable. Another limitation of this study is the reuse of the specimens, both the grafts and cemented femoral condyles. Reusing the graft after it has already been tested and fixated on the tibia via screws may have led to some compromising of the tissue itself. The additional cycling will also introduce some additional creep between the tests. Besides, due to the lack of freedom of the varus/valgus moment, a slight deviation of varus/valgus positioning may have resulted in an unequal load between the medial and lateral compartments when putting and positioning the knee into the knee simulator. Moreover, this controlled laboratory experiment cannot simulate muscle load, and we conducted an extensive soft tissue dissection to the posteromedial capsule in order to insert the Tekscan stress sensors, which may be different from the in vivo research. Although all of the conditions mentioned above may affect the knee joint contact area and stress, the conclusions of this study remain valid; the main purpose was to observe the biomechanical variations of the two ACLR conditions within each specimen.

## Conclusions

SB ACLR by the use of the AM portal method and TT method both alter the tibiofemoral contact area and stress when compared with the intact knee. When compared with the TT technique, ACLR by the AM portal technique more closely restores the intact tibiofemoral contact area and stress at low flexion angles.

## Abbreviations

ACL: Anterior cruciate ligament
ACLR: Anterior cruciate ligament reconstruction
AM: Anteromedial
SB: Single-bundle
TT: Transtibial
